# Megavoltage X-ray Dose Enhancement with Gold Nanoparticles in Tumor Bearing Mice

**Published:** 2013

**Authors:** Sayyed Hossein Mousavie Anijdan, Seyyed Rabi Mahdavi, Alireza Shirazi, Mohammad Ali Zarrinfard, Jamshid Hajati

**Affiliations:** 1 *Department of Medical Physics, Babol University of Medical Sciences, Babol, Iran.*; 2 *Department of Medical Physics and Engineering, School of Medicine, Tehran University of Medical Sciences, Tehran, Iran.*; 3 *Department of Medical Nanotechnology, Faculty of Advanced Medical Sciences, Tehran University of Medical Sciences, Tehran, Iran.*; 4 *Department of Immunology, Faculty of Medicine, Tehran University of Medical Sciences, Tehran, Iran.*

**Keywords:** Gold nanoparticles (GNPs), radiation dose enhancement, tumor, megavoltage X-ray

## Abstract

One of the applications of gold nanoparticles (GNPs) in medicine is radiation dose-enhancing effect. Although there are many simulations, *in vitro* and *in vivo* evidence that GNPs can enhance significantly the radiation dose effect of orthovoltage beams. These beams compared with megavoltage (MV) beams, have limited applications in radiotherapy. In order to evaluate GNPs radiosensitization performance with MV beams *in-vivo*, we used two most clinically used X-ray beams (6 and 18 MV) with the dose of 20 Gy for each mouse. Intratumoral injection of 50 nm GNPs with the concentration of 5 mg ml^-1^ was applied to melanoma tumor growing in the left leg of 7 to 8 mice in 4 control and treatment groups of C57BL/6 mice. Albeit, using 10 cm plexiglass jig phantom in the beam path improved the radiation - treatments, the statistical differences between groups were not significant. According to the results, it is concluded that mice can be treated with smaller tumors and higher concentrations of GNPs in MV radiation beams.

Radiotherapy is an important method in cancer treatment, but it can be held responsible for biological damages as radiations can also induce a decrease in normal tissues. Improvements in this cancer therapeutic method have begun in the past decades by performing intensity modulated radiation therapy (IMRT), multileaf collimators (MLCs) and stereotactic radiosurgery. Radiosen-sitization of tumors with safety media is an alternative method to improve the discrimination between tumors and normal tissues (1-3). Initially, this technique was known as X-ray phototherapy with iodine and gadolinium contrast media (4-5). Then it was introduced as contrast-enhanced radiation therapy or CERT (6-7) but in numerous papers, this method was mentioned as gold nanoparticle radiation therapy or GNRT (8-9). In recent years, this method has been of an increas-ingly interest by the use of gold nanoparticles radiosensitize contrast media due to its high atomic number and bio-compatibility (8). Through the progress in nanotechnology, the synthesis of various gold nanostructures such as spherical gold nanoparticles (GNPs), gold nanorods (GNRs), gold nanoshells (GNSs) and gold nanocages (GNCs) in cancer therapeutics have been made (2). Gold nanostructures can be applied as radiation sensi-tizers, anticancer drug enhancers, heat generators and also effective drug carriers.

The first step of this study was a series of computer simulations (10). The Monte Carlo method was described in several studies in the last years. These studies have all shown the radiosensitization of GNPs, especially with kilovoltage radiation exposures (11-14). But recent studies have also shown the radiosensitization effect at megavoltage beams (15-17). This effect can be understood on the basis of Local Effect Model (LEM) at nanometer scale.

Many other *in vitro* studies have also demonstrated dose enhancement with different cells at low voltage X-rays (18-22). Jain et al. (2010), for instance, performed clinically relevant MV X-ray with 2 nm GNPs and obtained comparably the same effect at kilovoltage (23). A recent *in vitro* study has shown radiation sensitizer enhancement ratios (SERs) of 1.29 and 1.16 at using 6 MV and 15 MV, respectively (24). Another recent study has indicated the relative biological effectiveness of proton beam on prostate tumor cells, approximately 15%-20% radiosensitization when cells contain internalized 44 nm of GNPs (25).

Animal studies have also shown that gold microspheres or nanoparticles can enhance radiobiological effect of radiation dose (18, 26-29). In some of these studies with the enhanced permeation and retention (EPR) effect, nanoparticles can accumulate in tumor through passive targeting (30). Hainfeld et al. were the first researchers who successfully demonstrated long time animal study with relatively high concentration 2 nm GNPs passively (26). Apoptotic potential and dose-enhancing effect of clinical electron beams were investigated on B16F10 melanoma tumor-bearing mice (27). Therefore, in this study, we investigated the dose-enhancing effect of gold nanoparticles in combination with single-dose clinical 6 and 18 MV photon beams on B16F10 melanoma tumor-bearing mice.

## Materials and Methods

**Cells and mice**

**Fig. 1:**
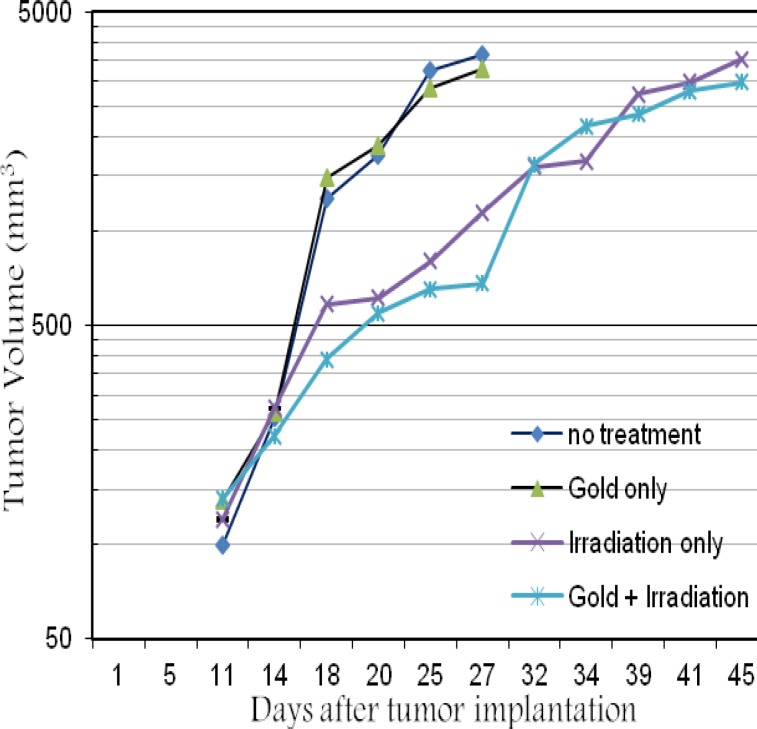
Average tumor volume at different groups of mice irradiated with 18 MV after: no treatment (diamonds, PBS); gold only (triangles); irradiation only (cross); intratumoral gold injection followed by irradiation (stars).

One T_25 _flask of murine B16F10 melanoma cells was obtained from National Cell Bank of Pasteur Institute of Iran (NCBI, C542). These cells were cultured in RPMI 1640 (Gibco G002909-2037) medium supplemented with 2 nM L-glutamine, Penn/ Strep (100 U ml^-1^ Penicillin; 100 μg ml^-1^ Streptomycin) and fetal bovine serum (Gibco A15111-0229) at 37 ^º^C in a pressure of 5% CO2. After two or three subcultures, the cells were prepared for injection into the mice. Female C57BL/6 mice (8-10 weeks old) were also obtained from the Karaj Production and Research Center Laboratory of Animal Science Department of the Pasteur Institute of Iran. All the experiments performed on mice were also approved by the Animal Care Committee of Tehran University of Medical Sciences. These C57BL/6 mice were planted subcutaneously (s.c.) in the thigh with 150000 murine isogenic melanoma B16F10 cultured cells suspended in PBS in a total volume of 40-50 μl. After 2 weeks, the tumors volume reached about 400-600 mm^3 ^(Fig. 1).

Tumor volume was estimated by measuring three diameter of mass and calculated with orthogonal diameters equation (31). One group of tumor bearing mice (including eight mice) had direct intratumoral injection with 100-200 μl of 5 mg ml^-1^ GNPs. All mice were monitored for the growth of tumor and survival during the experiments.

**Gold nanoparticles and biodistribution**

50 ± 2 nm gold nanoparticles (Plasmachem, Germany) were injected intratumorally at several sites. The volume level of GNPs injected to mice was in accordance with their accumulation in tumor proposed by Hainfeld et al. (26, 28). The GNPs concentration was about 7 mg ml^-1^ in the tumor. Digital radiographic images of the mice were used to identify intratumoral distribution of GNPs.

**Characteristics of irradiation**

The mice were anesthetized with Xiluzin and Ketamin intraperitoneally. About 10 mm in diameter of the region of mice leg containing the tumor was irradiated with 6 and 18 MV X-rays through Varian 2100 C/D linear accelerator (LINAC) approximately 30 min post GNPs injection. The output rate was 300 MU/min and the total delivered dose was 20 Gy.

**Fig. 2:**
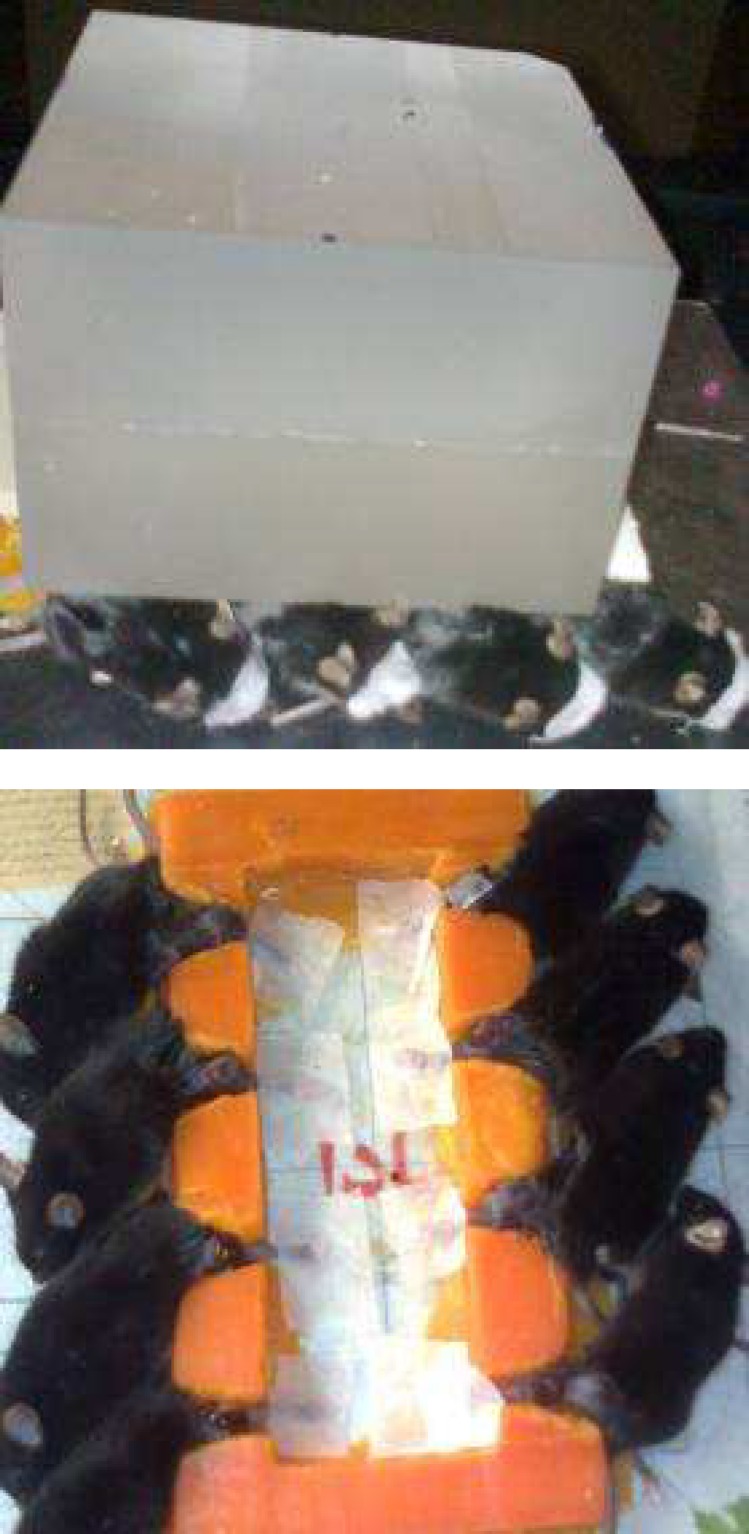
Set-ups of mice irradiation with plexiglass phantom and wax formed slab

It was not possible to perform this high single fraction dose to this depth tumor with LINAC for each mouse separately. Therefore a special phantom was designed that consisted of two separate slabs. The upper slab with the thickness of 10 cm plexiglass is thick enough to produce transient charged particle equilibrium for 6 and 18 MV X-ray beams. The lower slab was made from wax with eight places for eight tumor bearing legs of mice (Fig. 2). Radiation was applied to an 8.5 cm × 20 cm field size at 108 cm source-to-surface distance (SSD). The total 20 Gy dose was delivered with five 576 MU’s separated very short intervals. The time within each fraction was at least 5 minute.

**Fig. 3:**
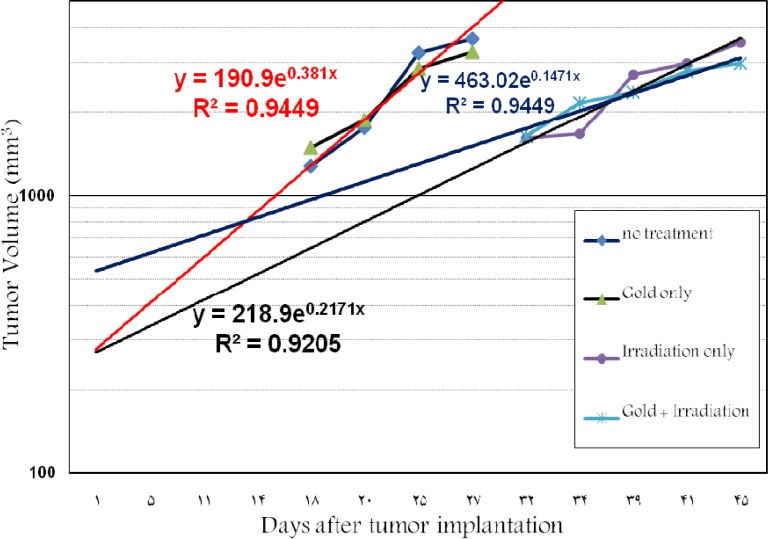
Tumor volume doubling time at different groups of mice irradiated with 18 MV

## Results

B16F10 melanoma cells were cultured and grown subcutaneously in the left legs of the C57BL/6 mice. The growth of the tumor and mice survival were also considered indices for the different treatments groups. Figure 1 shows the average tumor volumes of the different groups of mice irradiated with 18 MV. As shown in this figure, the rate of tumor size in control tumor bearing mice (no treatment and gold only) was different with the group receiving radiation only and the mice with GNPs and radiation. The average tumor volumes after various treatment modalities were meaningfully different between control groups and treatment groups. But this difference is not significant between irradiation only and gold with irradiation groups (P>0.05). It is depicted that in the first month after irradiation, tumor size control in gold with irradiation groups was better than irradiation groups only.

Figure 3 shows the tumor volume doubling time for three groups. Growth speed of tumor is identified from the slope of these lines as 0.381, 0.246 and 0.147 for the three groups 2-4. The volume doubling time were obtained 44, 76 and 113 hr for groups 2-4, respectively.

**Fig. 4:**
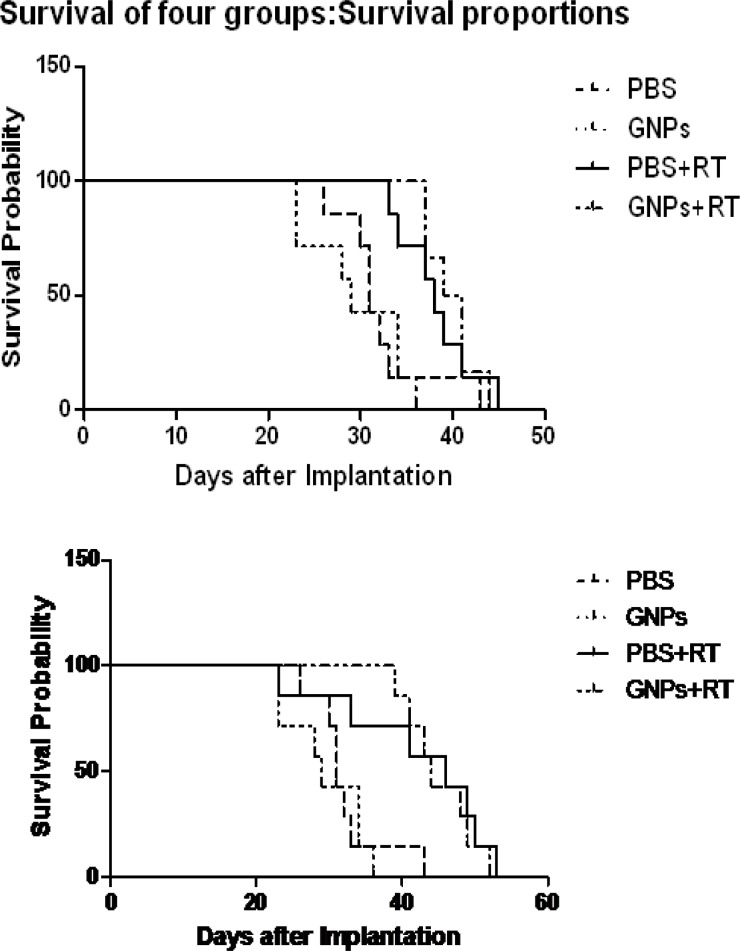
Kaplan-Meier survival graph of mice with different treatments, with 6 MV (up) and 18 MV (down).

Kaplan-Meier survival graphs (Fig. 4) have also shown the differences between groups. The GNPs without radiation could not stop the growth of the tumor and increased the survival of the mice. All mice receiving either no radiation or GNPs without radiation died during the span of one month.

## Discussion

In this study, *in vivo* GNPs radiosensitization was evaluated in radiotherapy with clinical megavoltage X-ray beams. We used the same geometrical set-ups utilized in the separate simulation and dosimetric study (10). DEF between 8-10% was obtained in deep tumor-like insert phantom (10 cm depth) that contained GNPs and irradiated with 18 MV.

It appears that the size of tumor is a very important parameter in radiotherapy. This study is one of the novel works that deals with high energy X-ray beams. It is revealed that tumor size and its response to the various treatments determine the shape of cell survival probability. We carried out treatments with about 500 mm^3^ tumor size while Hainfeld and Chang performed their *in vivo* studies with very small tumor sizes (100 mm^3^ approxi-mately) (26-27). Another motive was GNPs desired concentration in the tumor. Thus, tumor size was only controlled in the first month after irradiation with 18 MV (Fig. 1). This effect occurred much less in 6 MV. As seen in figure 3, the tumor size doubling time shows the topic. Because GNPs were injected intratumorally, the issue of the high liver uptake was not important for GNPs with 50 nm (26). Results reported from the MC simulation studies have shown that nanoparticles greater than 50 nm provide a more significant dose enhancement (12). An *in vitro* study by Chithrani et al*.* shows that cellular uptake is maximized when the size of nanoparticles is 50 nm (19). However, direct intra-tumoral injection has the potential disadvantage of non-uniform distribution, but in this study the GNPs were injected in several directions and sites in the tumor (18).

Jain et al. conducted experimental studies with 2 nm gold nanoparticles and observed a comparable dose enhancement in megavoltage similar to the kilovoltage CERT (23). They concluded that the increased X-ray absorption was not the main mechanism of physical dose enhancement. Also, it can be considered that there is a discrepancy between the physical dose enhancement in X-ray energy deposition and biologically observed dose enhancement in cell killing with different GNP sizes that are explained below (32). Usually, MC simulations consider GNP as a single particle and calculate the deposited energy with the radiation interaction. If the sizes of the GNPs are small (e.g. 2 nm), the interaction frequency and dose enhancement will be small and vice-versa. Also the results of Leung et al. show that GNP with greater sizes increases the generation of secondary electrons. However, inside the cells, small-sized GNPs form a single large cluster within the vesicles with a size of about 300-500 nm (12).

A new theoretical approach in calculating radiosensitization is the microscopic dose enhancement that is related to the deposited dose on the walls of tumor blood vessels (15, 33-34). Berbeco et al. revealed a 1.7 endothelial dose enhancement factor (EDEF) after the application of 30 mg ml^-1^ intravascular GNPs concentration for 6 MV beam at the depth of 20 cm (17). Furthermore they reported high EDEF values of 1.2 to 4.4 (24-340% dose increase) that happen in the vicinity of the nanoparticles. These findings also help us to explain the DEF discrepancy between our results and Robar and Cho (5, 8). Contribution of 10 cm slab phantom above the GNPs container should be considered to produce low energy X-rays and scattered radiation from 6 and 18 MV primary incident photons which cause dose enhancement within the tumor immediately after GNPs through appropriate interaction mechanisms.

Our study has shown that local dose enhanc-ement can be achieved by the GNPs with 6 and 18 MV photon beams at the depth of 10 cm. It also shows that the method of nanoparticles distribution can effectively change the DEF. In spite of all relevant dosimetric and simulation results that confirm dose enhancement, more research is suggested to be done for more biological clue. To achieve biological evidence, additional *in vivo* experimental set ups similar to this study should be conducted to investigate as to whether gold nanoparticles have the potential to be used as a radiosensitizer in radiation therapy of tumors.
